# Surveying DNA Elements within Functional Genes of Heterocyst-Forming Cyanobacteria

**DOI:** 10.1371/journal.pone.0156034

**Published:** 2016-05-20

**Authors:** Jason A. Hilton, John C. Meeks, Jonathan P. Zehr

**Affiliations:** 1 University of California Department of Ocean Sciences, Santa Cruz, California, United States of America; 2 University of California Department of Microbiology and Molecular Genetics, Davis, California, United States of America; CEA-Saclay, FRANCE

## Abstract

Some cyanobacteria are capable of differentiating a variety of cell types in response to environmental factors. For instance, in low nitrogen conditions, some cyanobacteria form heterocysts, which are specialized for N_2_ fixation. Many heterocyst-forming cyanobacteria have DNA elements interrupting key N_2_ fixation genes, elements that are excised during heterocyst differentiation. While the mechanism for the excision of the element has been well-studied, many questions remain regarding the introduction of the elements into the cyanobacterial lineage and whether they have been retained ever since or have been lost and reintroduced. To examine the evolutionary relationships and possible function of DNA sequences that interrupt genes of heterocyst-forming cyanobacteria, we identified and compared 101 interruption element sequences within genes from 38 heterocyst-forming cyanobacterial genomes. The interruption element lengths ranged from about 1 kb (the minimum able to encode the recombinase responsible for element excision), up to nearly 1 Mb. The recombinase gene sequences served as genetic markers that were common across the interruption elements and were used to track element evolution. Elements were found that interrupted 22 different orthologs, only five of which had been previously observed to be interrupted by an element. Most of the newly identified interrupted orthologs encode proteins that have been shown to have heterocyst-specific activity. However, the presence of interruption elements within genes with no known role in N_2_ fixation, as well as in three non-heterocyst-forming cyanobacteria, indicates that the processes that trigger the excision of elements may not be limited to heterocyst development or that the elements move randomly within genomes. This comprehensive analysis provides the framework to study the history and behavior of these unique sequences, and offers new insight regarding the frequency and persistence of interruption elements in heterocyst-forming cyanobacteria.

## Introduction

Cyanobacteria can alter their cell shape and size, cell wall thickness, and filament orientation in response to environmental conditions varying from nutrient limitation to predation [1–3]. The morphological diversity of cyanobacteria also extends to cell differentiation as some filamentous cyanobacteria can form akinetes, hormogonia, and heterocysts, in addition to vegetative cells. Akinetes are spore-like cells formed during unfavorable growth conditions [4], while hormogonia are small-celled, motile filaments that are especially important in symbiosis initiation [5]. Heterocysts are the sites of N_2_ fixation under nitrogen (N) limitation [6].

N_2_ fixation, the process of converting N_2_ to NH_3_, can be a means of avoiding a common nutrient limitation for microbial growth in a variety of environments [7,8]. Nitrogenase, the enzyme that catalyzes N_2_ fixation, is sensitive to inactivation by oxygen (O_2_) [9], and must withstand the O_2_ that is in the environment and that evolves through photosynthesis. Heterocysts create and maintain a microoxic microenvironment by shutting down the activity of O_2_-evolving photosystem II, increasing respiratory O_2_ uptake, and creating a thick envelope around the cell wall to restrict gas diffusion [6].

**Fig 1:**
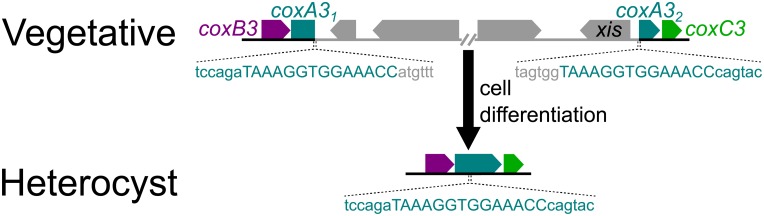
The *coxA3* element found in the *Calothrix* sp. PCC 7103 genome. An example of an element interrupting a gene (split into *coxA3*_*1*_ and *coxA3*_*2*_) in the vegetative genome. The complete, functional *coxA3* gene is present in the heterocyst genome due to the element excision by the protein encoded by the *xis* gene.

Heterocyst differentiation is regulated by many signaling pathways [10] and is accompanied by the changes in the expression of 500 to 1,000 genes [11,12]. In some heterocyst-forming cyanobacteria, genome rearrangement is also necessary in order to form fully functional heterocysts. Key N_2_ fixation genes are often interrupted in the genome of vegetative cells by DNA sequences, referred to herein as interruption elements, previously observed to be as long as 80 kb in length [13]. Excision of an interruption element during heterocyst formation results in a contiguous, functional gene in heterocyst genomes [14] (Fig 1). These elements are not in all heterocyst-forming cyanobacterial genomes, but have been commonly found within *hupL*, which encodes one subunit of an uptake hydrogenase responsible for recycling hydrogen (H_2_) produced during N_2_ fixation, and *nifD*, which encodes the nitrogenase alpha subunit [13–18]. Additionally, elements have been reported within *fdxN*, which encodes a ferredoxin and is located in an operon with nitrogenase genes *nifU*, *nifS*, and *nifB*, and likely *nifH*, *nifD*, and *nifK*, as well. [14,15,17,19,20]. More recently, elements have been found in the nitrogenase iron subunit gene *nifH*, and in *nifK*, the nitrogenase beta subunit gene [13,21,22].

The elements are removed during the later stages of heterocyst development by a site-specific recombinase, which is encoded by a gene located within the element [15,23]. To excise the interruption element, the recombinase binds to direct repeats that bracket the element [24]. Genes for these recombinases have been identified as *xisA*, *xisC*, and *xisF* within *nifD*, *hupL*, and *fdxN* elements, respectively [14,25,26]. The *nifH* element recombinase gene sequences is most similar to that of *xisA* [21], and the sequence of the recombinase gene in the *nifK* element is most closely related to the *xisF* sequence [22]. *xisA* expression is likely regulated by the transcriptional regulators for nitrogen, NtcA, and ferric uptake, FurA; however, little is known of the details of the regulatory mechanisms and other potential regulating factors of interruption element recombinases [27–29]. Two additional genes, *xisH* and *xisI*, located in the *Nostoc* sp. strain PCC 7120 (*Nostoc* 7120) *fdxN* element are also required for excision of that element [30]. The specific roles of *xisH* and *xisI* are unclear, but they may be related to determining the direction of recombination catalyzed by XisF—integration vs. excision [31]. It is also unclear when or how all of these interruption elements originated; it has been hypothesized that they are the remnants of viruses or phages [18,32].

Yet another mystery is whether the elements provide any advantage or disadvantage to heterocyst-forming cyanobacteria or if they are selfish DNA, and have little to no effect(s) on the organisms [33]. The possession or lack of a *nifD* element did not affect cell growth or N_2_ fixation of two different *Nostoc* strains in the presence of combined N or with N_2_ as the sole N source [34,35]. Thus, the presence or absence of this element has no apparent selective advantage under laboratory growth conditions, at least not within the timescale of these experiments.

In an effort to more fully explore the evolution and potential purposes of the interruption elements, the work presented here examines the elements found in all currently sequenced heterocyst-forming cyanobacterial genomes. We defined 101 interruption elements in heterocyst-forming cyanobacterial genomes, including many previously unknown elements, and compared them in an attempt to determine their evolutionary paths. Additionally, in an effort to provide evolutionary context for these elements, we searched all cyanobacterial genomes for interruption elements and discovered three elements within genes of cyanobacteria that are not capable of forming heterocysts.

## Materials and Methods

**Fig 2:**
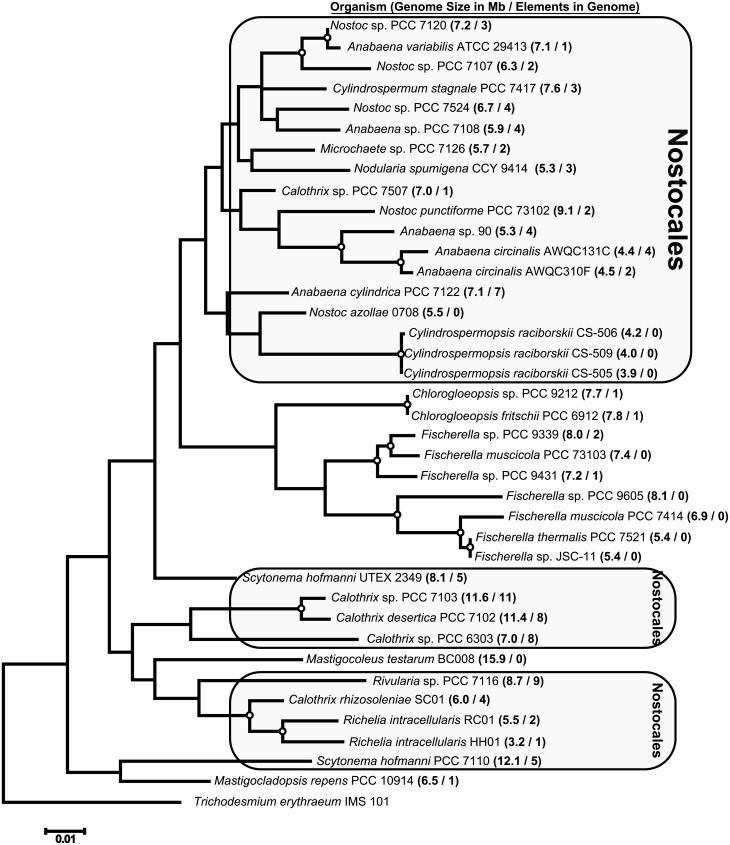
Heterocyst-forming cyanobacteria 16S phylogeny. Maximum likelihood phylogenetic tree of 16S rRNA from 38 heterocyst-forming cyanobacterial genomes, rooted with *Trichodesmium erythraeum* IMS 101. The size of the genome and the number of interruption elements within the genome are shown within parentheses. Cyanobacteria of the order Nostocales are in shaded boxes, and all other cyanobacteria belong to the order Stigonematales, with the exception of *T*. *erythraeum* IMS101. Open circles at branch connections indicate a bootstrap value of at least 75%.

As of the writing of this report, there were 38 heterocyst-forming cyanobacteria with sequenced genomes; 27 from the order Nostocales and 11 from the order Stigonematales (Fig 2). The *Calothrix rhizosoleniae* SC01 genome is yet unreleased, but sequences relevant to this study can be found in S1 and S2 Data. Accession numbers for all of the other genomes can be found in Table A in S1 File. Annotations of the genomes were examined, focusing on regions near the five genes previously shown to contain interruption elements (*nifH*, *nifD*, *nifK*, *hupL*, and *fdxN*). The gene synteny surrounding each of these genes is highly conserved in heterocyst-forming cyanobacteria. Any genes found between *nifH*, *nifD*, *nifK*, *hupL*, or *fdxN* and the expected adjacent gene were examined for the presence of a gene with similarity to one of the known *xis* genes. A BLAST analysis (blastp) was conducted with these recombinase genes against all protein sequences of the 38 genomes. Each gene with a hit to any of the *xis* genes (with an *in silico*-determined threshold e-value ≤10^−20^) was examined as follows. A BLAST analysis (blastx, e-value ≤10, maximum matches in a query range = 15) was conducted for each *xis* gene candidate and surrounding nucleotides (3 kb up- and down-stream) against the nr database (NCBI). Genes that were similar to either region flanking the candidate *xis* gene, but truncated, were used as references for the possible interrupted gene. We then used each reference gene as a query to a BLAST search (tblastn, e-value ≤10) against the entire genome in order to locate sequences similar to different sections of the reference gene. Those genome regions were then aligned with the reference gene to confirm that they formed an interrupted gene. Once aligned, the direct repeats were identified in the overlapping bases of the two genome regions (Fig 1). We did not allow for any mismatches to occur between direct repeats, and thus, these may not represent the entire target sequence, but for the purposes of this study, serve as a marker for the end and beginning of each element. As more interruption elements were confirmed, the *xis* gene sequences from each were added to the set, and the BLAST analysis was repeated to find more potential gene interruptions and associated *xis* genes.

A BLAST analysis was then conducted with all of the confirmed *xis* genes from the heterocyst-forming cyanobacterial genomes against all non-heterocyst-forming cyanobacterial genomes (blastp, e-value ≤10^−50^). More stringent criteria was used for this BLAST analysis in order to limit our results to only those recombinase genes most similar to the known *xis* genes. All BLAST hits were checked for interrupted genes as described above.

For the recombinase gene and 16S rRNA phylogenetic trees, nucleotide sequences were aligned in MEGA5 with ClustalW [36]. The recombinase gene sequence alignments were done with respect to codons, and thus, no gaps were inserted within a codon. Maximum likelihood phylogenetic trees were constructed with MEGA5 using the Tamura-Nei model and only those alignment sites with at least 90% coverage were used [37]. Statistical support for nodes was based on 1,000 bootstrap replicates [38].

To analyze shared genes within the gene content of the elements, a bi-directional BLAST analysis (blastp, e-value ≤10^−20^) was conducted on all of the genes from each heterocyst-forming cyanobacterial genome against the full gene set from each of the other genomes. We then identified the genes encoded in an element that had bi-directional best hits to at least four other genes from elements. The bi-directional best hits of each of those genes were then used to identify non-element genes occurring in other genomes.

## Results

We identified 101 interruption elements within 28 genomes of heterocyst-forming cyanobacteria, while an additional 10 genomes did not have any observed elements (Fig 2). Five out of 11 cyanobacteria within Stigonematales each had at least one element. On the other hand, 23 out of 27 Nostocales organisms possessed at least one interruption element, with as many as 11 elements in one genome (*Calothrix* sp. PCC 7103). The structure of the phylogenetic tree generated based on the 16S rRNA sequences of these 38 genomes largely agreed with phylogenetic trees that were made using concatenated gene sequences from many of the same genomes, with the main exception being *Anabaena* sp. PCC 7108 which has grouped more closely with *Anabaena cylindrica* PCC 7122 and *Nostoc azollae* 0708 in other trees [39,40].

The interruption elements were as small as the 1.3 kb element within a *Scytonema hofmanni* UTEX 2349 FAD-dependent oxidoreductase gene up to the nearly 1 Mb *coxA* element in *Calothrix* sp. PCC 6303 (Table B in S1 File). The average element length was 41.5 kb, excluding seven elements that could not be characterized due to apparent genome mis-assembly and nine elements that were predicted from sequences on separate contigs, and thus may be longer than determined by genomic analysis. All of the elements that were found in this study and their *xis* genes are catalogued fully in Table B in S1 File.

The elements within heterocyst-forming cyanobacterial genomes interrupted 87 individual genes, and 10 of those genes were interrupted by multiple elements. The *Rivularia* sp. PCC 7116 *nifD* was interrupted by four different element, the most found within a single gene (Figure A in S2 File). Each of the 87 interrupted genes belonged to one of 22 different ortholog groups, but not all elements within a given ortholog interrupted the gene at the same location (Table 1). We considered all elements that interrupted the same ortholog at roughly the same position along that ortholog (± 10 bp) to represent an element variant. A slight fluctuation in the start positions of direct repeats within a variant was due to the varying lengths of the direct repeat. The 34 *nifD* elements belonged to four different element variants, while six element variants were found amongst the 19 *nifH* elements. Two variants each were found for *nifK*, *nifB*, *hupL*, *hupS*, and *coxA3* elements.

**Table 1:** The 22 ortholog groups that were found to have at least one representative gene interrupted by an element in heterocyst-forming cyanobacteria, and three additional orthologs that had interruption elements in non-heterocyst-forming cyanobacteria.

Gene Product	Gene Symbol	Reference Locus Tag	Elements	Variants
nitrogenase molybdenum-iron protein alpha subunit	*nifD*	Aazo_1353	34	4
nitrogenase iron protein	*nifH*	Aazo_1354	19	6
uptake hydrogenase large subunit	*hupL*	Aazo_3865	11	2
nitrogenase molybdenum-iron protein beta subunit	*nifK*	Aazo_1352	8	2
uptake hydrogenase small subunit	*hupS*	Aazo_3866	4	2
ferredoxin	*fdxN*	Aazo_1357	3	1
nitrogenase cofactor biosynthesis protein	*nifB*	Aazo_1358	2	2
nitrogenase molybdenum-iron cofactor biosynthesis protein	*nifE*	Aazo_1350	2	1
pyruvate ferredoxin/flavodoxin oxidoreductase	*nifJ*	Cal7507_5433	2	1
flavin reductase domain-containing FMN-binding protein	*flv3B*	Aazo_4140	2	1
cytochrome c oxidase subunit I	*coxA*	Aazo_2640	3	3
polyketide-type polyunsaturated fatty acid synthase	*hglE*	Aazo_3917	1	1
NADPH-dependent FMN reductase		Aazo_5221	1	1
FAD-dependent oxidoreductase		Cal7507_5656	1	1
phospholipase D/transphosphatidylase		Cal7507_0570	1	1
primase P4		Ana7108_2845	1	1
arabinose efflux permease		Mic7126DRAFT_5075	1	1
integrase family protein		Aazo_2682	1	1
transposase		Ava_B0242	1	1
transposase (ISSoc8)		Mas10914DRAFT_5058	1	1
caspase domain-containing protein		Cal7103DRAFT_00047390	1	1
hypothetical protein		CylstDRAFT_1988	1	1
**In non-heterocyst-forming cyanobacteria**				
predicted integral membrane protein		Pse6802_3453	1	1
hypothetical protein		Cya7822_6696	1	1
ATP-dependent DNA helicase	*recQ*	Pse6802_0098	1	1

The presence of element variants with more than three occurrences in the heterocyst-forming cyanobacterial genomes was plotted along the 16S rRNA phylogeny (Figures B and C in S2 File). The variants range from those organisms possessing them clustering together (e.g. *nifH* 405 and *fdxN*) to those that were spread out throughout the 16S rRNA phylogenetic tree (e.g. *hupL* 590 and *nifD* 895).

A *xis* gene was identified for 100 out of the 101 elements (Table B in S1 File). A contig break prevented the confirmation of a recombinase-coding gene in the *Richelia intracellularis* RC01 *nifH* element. There were also seven cases that involved the two sections of the interrupted gene oriented in opposite directions (Figure D in S2 File). These are likely due to mis-assembly of the genome, and have been labeled as "odd assembly" in Table B in S1 File. The sequences for the corresponding *xis* genes were included in phylogenetic analysis but no further characterization of the interruption elements was done. There were 44 additional genes with a BLAST hit to a *xis* gene (e-value <10^−20^), but an interrupted gene could not be located near them, and, thus, could not be verified as element excision genes, possibly due to the bias introduced by limiting our search for an interrupted gene to within 3 kb of each possible *xis* gene.

Of the *xis* genes found in this study, 48 encoded recombinases belonging to the serine recombinase superfamily, while the remaining 52 encoded recombinases of the tyrosine superfamily (Table B in S1 File). At least one element containing a serine recombinase was found interrupting each of the 22 ortholog groups, but only six ortholog groups had an occurrence of an element containing a tyrosine recombinase. The 23 *xis* genes aligned in the same orientation as the interrupted genes were located towards the beginning of the element. All but one of the 70 *xis* genes that were oriented in the opposite direction to the interrupted gene were found closer to the end of the element (example: Fig 1). The *xis* gene in the *Calothrix* sp. PCC 6303 arabinose efflux permease interruption element was the exception to this, and although oriented in the opposite direction of the interrupted gene, it was located at the beginning of the element.

**Fig 3:**
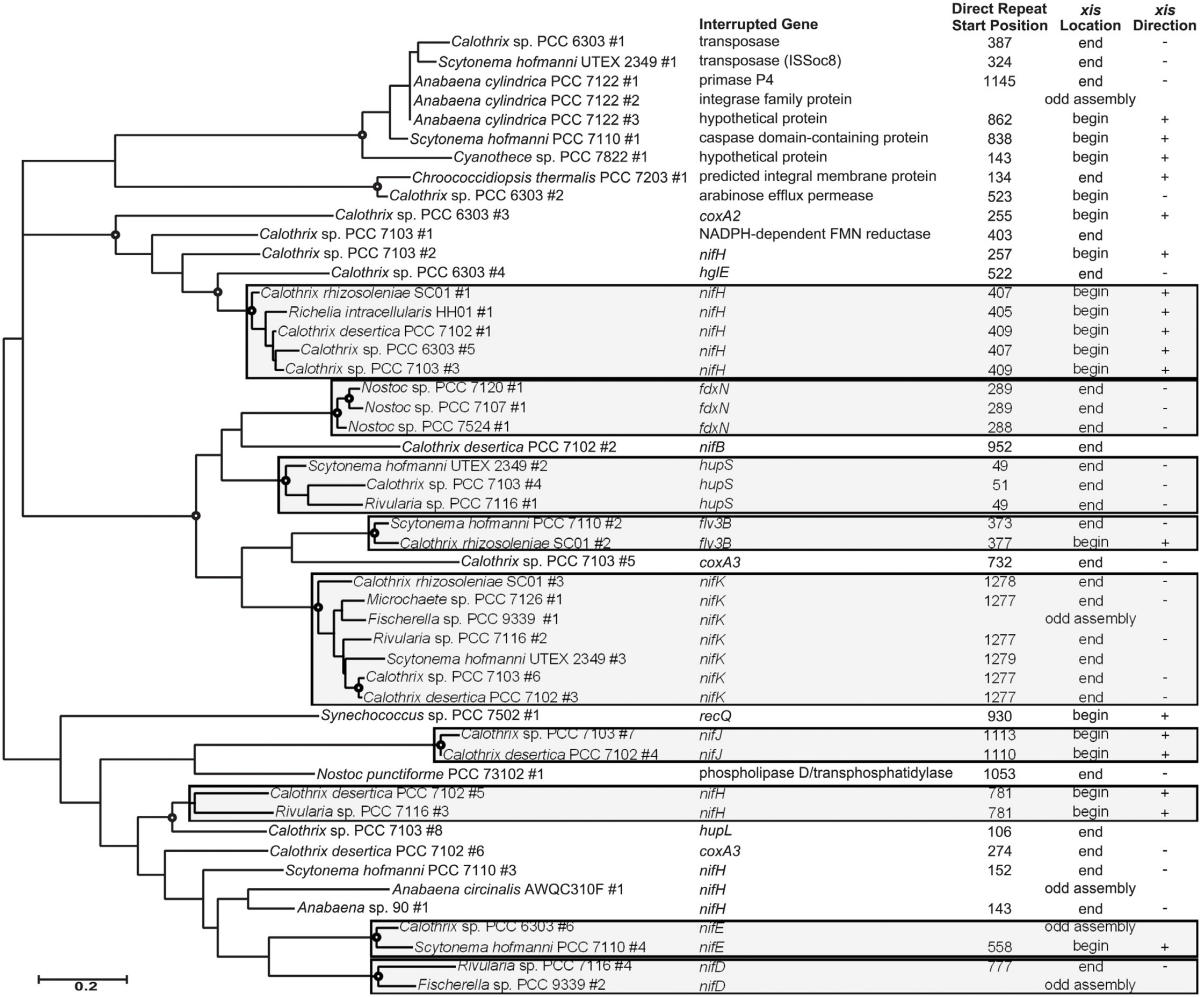
Serine recombinase phylogeny. Maximum likelihood phylogenetic tree of serine recombinase gene (*xis*) nucleotide sequences and data characterizing the element each *xis* gene is found on. Shaded boxes group element variants together. Open circles at branch connections indicate a bootstrap value of at least 75.

**Fig 4:**
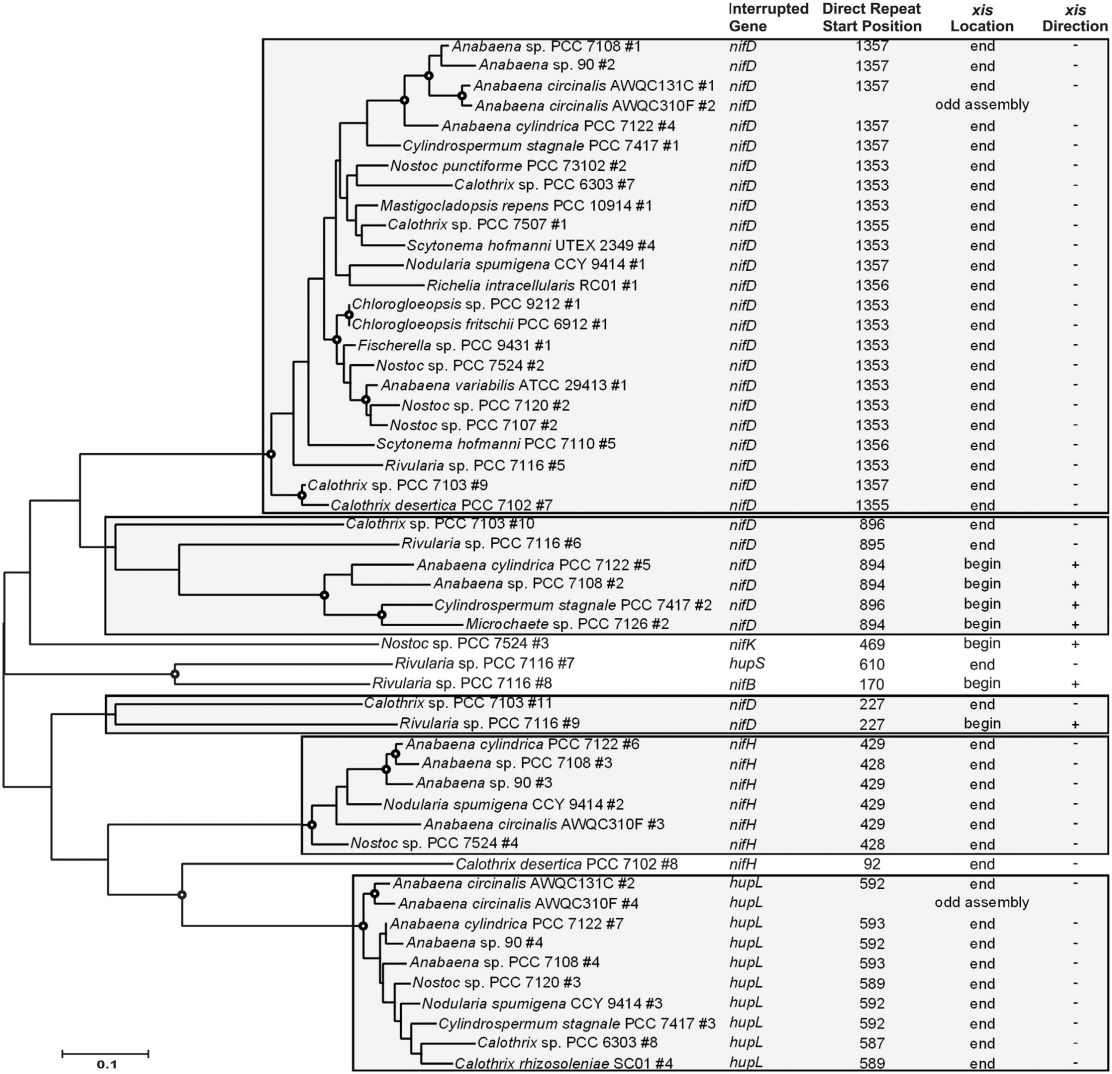
Tyrosine recombinase phylogeny. Maximum likelihood phylogenetic tree of tyrosine recombinase gene (*xis*) nucleotide sequences and data characterizing the element each *xis* gene is found on. Shaded boxes group element variants together. Open circles at branch connections indicate a bootstrap value of at least 75.

The majority of *xis* gene sequences formed well-supported phylogenetic clusters with other *xis* genes found in interruption elements of the same element variant (Figs 3 and 4), but there were a few exceptions. The three *xis* gene sequences of elements that interrupted *nifH* at position 150 (± 7 bp) were closely-related, but did not form a branch like the *xis* genes from each of the other variants (Fig 3; *Anabaena* sp. 90 element #1, *Anabaena circinalis* AWQC310F #1, and *Scytonema hofmanni* PCC 7110 #3). The *nifD* elements with insertion locations at position 895 (± 1 bp) contained *xis* gene sequences that clustered together, however, four of the genes were within a subcluster with strong bootstrap support (*Anabaena cylindrica* PCC 7122 #5, *Anabaena* sp. PCC 7108 #2, *Cylindrospermum stagnale* PCC 7417 #2, and *Microchaete* sp. PCC 7126 #2), while the *Rivularia* sp. PCC 7116 (#6) and *Calothrix* sp. PCC 7103 (#10) genes were phylogenetically distant (Fig 4). The *Rivularia* sp. PCC 7116 and *Calothrix* sp. PCC 7103 *xis* genes within these elements were located at the end of the element, oriented in the opposite direction as *nifD*, but the *xis* genes within the other four elements of this variant were found at the beginning of the element and read in the same direction as *nifD*. Similarly, the two *xis* gene sequences from the *nifD* elements with insertion locations at position 227 clustered together, but exhibited long branch lengths and low bootstrap support, and the *xis* genes differed in location and orientation (Fig 4; *Rivularia* sp. PCC 7116 #9 and *Calothrix* sp. PCC 7103 #11). The *flv3B* elements of *S*. *hofmanni* PCC 7110 and *Calothrix rhizosoleniae* SC01 also had *xis* genes of differing location and orientation (Fig 3). The *S*. *hofmanni* PCC 7110 *xis* gene was located at the end of the element and was oriented in the opposite direction of the *flv3B* that it interrupted, while the *C*. *rhizosoleniae* SC01 *xis* gene was at the beginning of the element and was read in the same direction as *flv3B*.

In some cases, *xis* gene sequences grouped phylogenetically by organism. For example, seven genes in the *A*. *cylindrica* PCC 7122 were identical to each other, and while no interruption element could be identified for four of the genes, the three other genes were identified as encoding element excision proteins (Fig 3; *A*. *cylindrica* PCC 7122 #1–3). Similarly, the *xis* gene of the interruption element within a *S*. *hofmanni* UTEX 2349 transposase was identical to the *xis* gene in an oxidoreductase gene interruption element in the same genome (Table B in S1 File). However, the oxidoreductase gene interruption element *xis* gene sequence was shortened by a gap in the genome assembly, and, thus, was left out of the analysis. Although more divergent than the *A*. *cylindrica* PCC 7122 and *S*. *hofmanni* UTEX 2349 *xis* genes, the *Rivularia* sp. PCC 7116 *xis* gene sequences responsible for the *nifB* and *hupS* elements clustered together (Fig 4). The length and GC content of each element was plotted along the *xis* phylogeny (Figures E-H in S2 File), but no clear patterns emerged.

Seven groups of genes were identified that were shared amongst at least five elements (Table C in S1 File). Four of these gene groups were shared amongst the *nifD* element variant that interrupts the gene at position 1355 (± 2 bp). These include a hypothetical protein that was found in 17 elements, as well as an ATPase gene that was found exclusively in elements. A hypothetical protein was conserved in five *hupL* elements and also found in one additional genome not in en element. A translation elongation factor G paralog was found in three different element variants of five genomes and in two other genomes, which have the potential to be in elements, as discussed below. A transposase gene was also found on multiple element variants.

Interruption elements were also found within genes of three unicellular non-heterocyst-forming cyanobacteria, including a hypothetical membrane protein of *Chroococcidiopsis thermalis* PCC 7203 (Table B in S1 File). A *Synechococcus* sp. PCC 7502 DNA helicase gene and a *Cyanothece* sp. PCC 7822 hypothetical protein were also interrupted by interruption elements. The *xis* gene sequences from these elements were spread throughout the serine recombinase gene phylogenetic tree, and all three genes were oriented in the same direction as the interrupted gene. The *xis* genes within the *Synechococcus* sp. PCC 7502 and *Cyanothece* sp. PCC 7822 elements were located at the beginning of their respective elements, while that of the *C*. *thermalis* PCC 7203 hypothetical protein element was the only positively oriented *xis* gene in this study to be found at the end of the element.

## Discussion

### Interrupted genes

The newly discovered interruption elements presented here provide context for which to answer many questions that have surrounded these features for decades. Among those orthologs that were not previously reported to contain elements are several genes known to be important to N_2_ fixation or maintenance of a microoxic microenvironment in heterocysts. The products of *nifE* and *nifB* are involved in the synthesis of the nitrogenase Fe/Mo cofactor [41], and *hupS* encodes an uptake hydrogenase subunit and is in an operon with *hupL*. *nifJ* encodes an oxidoreductase that is responsible for electron transfer to nitrogenase, and is required for N_2_ fixation when iron is limiting [42,43]. A previous study of *Nostoc* 7120 indicated that expression of genes orthologous to the two element-containing cytochrome c oxidase subunit I copies, *coxA2* and *coxA3*, are specific to heterocysts, while a third copy, *coxA1*, is expressed only in vegetative cells [44]. The role of cytochrome c oxidase in heterocysts is in respiratory O_2_ consumption [44]. Similarly, the *flv3B* gene product of *Nostoc* 7120 is a heterocyst-specific flavin reductase that reduces O_2_ [45]. The fatty acid synthase encoded by *hglE* is involved in the formation of heterocyst glycolipids, which form a layer in the envelope that acts to retard gas diffusion into the heterocyst [46]. Given the heterocyst-specific roles of these genes, the excision of their interruption elements is likely dependent on transcription of the respective *xis* genes during heterocyst formation, as is assumed to be the case with all of the previously reported elements [15].

The presence of interruption elements within genes with no known role in N_2_ fixation opens several possibilities. First of all, these interrupted genes may indeed have heterocyst-specific roles and are excised during heterocyst formation. Additionally, the elements may not be excised under any circumstances, thus, leaving the genes that they interrupt non-functional. However, this inactivation would likely lead to rapid deterioration of the interrupted gene, and likely the *xis* gene, as well. On the other hand, the excision of some elements could be brought on by factors other than heterocyst development, such as differentiation of other cell types. Many heterocyst-forming cyanobacteria also form akinetes [4] and hormogonia [5]. Additionally, the element-containing unicellular *Chroococcidiopsis* spp. form survival cells upon nitrogen limitation [47]; the excision of the interruption element from the gene encoding a membrane protein could be triggered by the formation of these cells. This idea is supported by a phylogenetic relationship between *C*. *thermalis* and heterocyst-forming cyanobacteria that provides a possible evolutionary link between these survival cells and heterocysts [40,48]. Thus, interruption element excision triggered by cell differentiation may be shared between these organisms.

The excision of an interruption element during differentiation of non-heterocyst cell types may require additional mechanisms for the element to persist. Excision of an element during development of a non-terminal cell type, such as hormogonia, akinetes, or the survival cells formed by *Chroococcidiopsis* spp., would require reintegration into the chromosome upon differentiation back into a vegetative cell. Tn3 family transposases are capable of integrating a donor sequence into a target sequence [24], and Tn3 family transposases are found adjacent to the recombinase genes of the interruption elements within the *Calothrix* sp. PCC 6303 arabinose efflux permease gene and the *C*. *thermalis* PCC 7203 membrane protein. Thus, each is a likely candidate to be excised from the chromosome in a non-terminal manner, and reintegration of these elements could be linked to the reason that the *xis* genes in these two elements are oriented in different ways than the others in this study. While element excision may be triggered by differentiation of various cell types, the mechanisms could differ depending on the cell type.

Factors other than cell differentiation should also be considered for triggers of interruption element excision in heterocyst-forming cyanobacteria. However, similar to the case of a non-terminal cell type, the excision of interruption elements throughout a population during a single event would result in the element being present in future generations exclusively as extrachromosomal DNA. Reintegration would again be required, but the interruption elements found in *Synechococcus* sp. PCC 7502 and *Cyanothece* sp. PCC 7822 do not contain transposases. The presence of interruption elements in these unicellular cyanobacteria, neither of which has been shown to differentiate cells of any kind, supports the possibility of other factors prompting genome rearrangements.

### Evolution of recombinase genes and element variants

The two recombinase superfamilies (tyrosine and serine) responsible for interruption element excision from heterocyst-forming cyanobacterial genes are named for the amino acid residue of each that covalently binds to the DNA, and each superfamily uses a different mechanism for recombination [24]. They have different evolutionary histories, and the lack of an evolutionary relationship between the recombinase superfamilies indicates there have been at least two events in which the heterocyst-forming cyanobacterial lineage has acquired interruption elements, one involving each type of recombinase.

The relatedness of the recombinase gene sequences within each superfamily provides clues as to how the interruption elements have evolved in heterocyst-forming cyanobacteria. In general, the recombinase gene sequences mostly clustered with those from the same element variant in other organisms. However, the *A*. *cylindrica* PCC 7122 and *S*. *hofmanni* UTEX 2349 sets of serine superfamily recombinase gene sequences clustered with others from the same organism, and are likely the result of transposition events within the genome. The *Rivularia* sp. PCC 7116 *xis* genes responsible for the *nifB* and *hupS* elements are the only examples of tyrosine recombinase gene sequences from the same organism clustering together, and they are much more divergent than those serine recombinase gene clusters. Additionally, serine superfamily recombinase genes are found in elements interrupting more ortholog groups than tyrosine superfamily recombinase genes, including all of the genes with no known N_2_ fixation role. The relationships of the available recombinase gene sequences provide evidence that serine superfamily recombinase genes have a greater tendency to integrate into a genome or replicon, or replicate within a genome than tyrosine superfamily recombinase genes. This replication is a characteristic of selfish DNA [33], and is likely the mechanism for the origin of an element variant.

The element variant that interrupts *nifD* at position 1355 (± 2 bp) was, by far, the most common variant observed in the genomes studied here. This was likely either the first interruption element to integrate and persist in heterocyst-forming cyanobacteria, or this particular element variant is more likely to be retained than other elements, possibly due to some advantage it provides to the host organism. Either way, it is this frequently occurring element variant that warrants special attention when examining the origin of interruption elements within this lineage.

### Element gene content

Genes present in multiple interruption elements linked some elements together, possibly through their shared origin, such as infecting phages that contained the conserved gene. We were able to identify several genes that were shared amongst elements of a single variant, most commonly the elements that interrupt *nifD* at position 1355 (± 2 bp), including several that have been previously identified as conserved in many of those elements [18,49,50]. Most of these genes were also found in genomes outside of interruption elements, indicating their functions are likely not element-specific despite their conservation in many elements. The ATPase that was found in six elements is the exception as it was not found in any other genome, although it is unclear what possible function it may have with regards to the element.

The genes encoding translation elongation factor G (TEFG) paralogs in multiple element variants also may have a function specific to the element. TEFG paralogs were found in the *Richelia intracellularis* HH01 and *R*. *intracellularis* RC01 *nifH* elements, the *Calothrix* sp. PCC 7103 and *Calothrix desertica* PCC 7102 *nifK* elements, and the *Rivularia* sp. PCC 7116 element that interrupts *nifD* at position 1353. The *C*. *rhizosoleniae* SC01 TEFG paralog is in an 11.9 kb contig, and may be in one of several interruption elements that are disjointed by a contig break (*hupL*, *nifH*, *nifK*, or *flv3B*) or not in any interruption element. Similarly, the *M*. *testarum* BC008 TEFG paralog is located in a 40.5 kb contig. No interruption element has been confirmed in that genome, but there are two possible *xis* genes that are located near contig edges, and thus, are possible element recombinase genes. Therefore, the TEFG paralog may be exclusively located in interruption elements in heterocyst-forming cyanobacteria. However, the interruption elements containing the TEFG paralogs are not closely related, based on recombinase gene sequences, indicating the TEFG paralog was not simply inherited with that interruption element through speciation. In bacteria, TEFGs are involved in elongation during protein synthesis and ribosome recycling [51–53]. In addition to a TEFG of cyanobacterial ancestry, a TEFG paralog is found in some cyanobacteria [54], including the seven closely related heterocyst-forming strains analyzed here. Thus, the TEFG paralog may be preserved in interruption elements due to an element-specific function, such as a role in the synthesis of proteins encoded in the excised element in the heterocyst.

Based on the relatedness of their recombinase genes, the interruption elements within each variant appear to have a strong evolutionary connection, yet there are very few physical similarities within variant groups. The lack of consistencies in length or GC content among all interruption elements of a single variant indicates that the interruption elements are dynamic sequences that have undergone changes within the individual organisms. The highly variable gene content encoded in each interruption element is also indicative of a dynamic sequence with gene deletions and insertions taking place over time. The transformations that the interruption elements have undergone over time have clouded genetic signatures and made it difficult to connect the elements to their origin.

## Conclusion

The extensive analysis reported here provides a framework for which to study interruption elements in cyanobacteria. Given the general lack of conserved gene content in the interruption elements, the recombinase gene sequences provide the best foundation for which to evolutionarily link the elements. A lack of homology between the two recombinase superfamilies implies at least two distinct origins and evolutionary paths of interruption elements in heterocyst-forming cyanobacterial ancestry. However, the relatedness of recombinase gene sequences within each superfamily sheds light on the origin of element variants through recombinase gene replication within a genome, and shows a greater tendency for serine superfamily recombinase genes to undergo this replication. While the previously reported *nifD* and *hupL* interruption elements are among the most frequently occurring elements in heterocyst-forming cyanobacteria, our analysis uncovered elements interrupting other gene orthologs, as well as variants of the interruption elements in previous reports. Our discovery of elements that are closely related to elements that have excision triggered by the cell, but interrupt genes that have no known function in N_2_ fixation, reveals the possibility of factors beyond heterocyst differentiation that could activate the excision of interruption elements. Through expansion of the interruption element data set, we have begun to trace the evolutionary paths of these unique genetic features and identified that their impact is broader than previously thought.

## Supporting Information

S1 Data *Calothrix rhizosoleniae* SC01 gene sequences. The DNA sequences for the four *xis* genes (from Table B in S1 File) and three genes that were shared across elements (from Table C in S1 File) in the *Calothrix rhizosoleniae* SC01 genome.
(FAS)

S2 Data *Calothrix rhizosoleniae* SC01 contig sequences. The seven contigs from the *Calothrix rhizosoleniae* SC01 genome that contained part of an element.
(FAS)

S1 File Tables A-C. Detailed information describing the genomes analyzed in this study, the interruption elements that were found in those genomes, and shared genes across the elements.
(XLSX)

S2 File Figures A-H. Images providing depictions of specific interruption elements, element frequency relative to phylogeny, and element length and GC content trends.
(PDF)
