# Supplementary material for: Surveying DNA Elements within Functional Genes of Heterocyst-Forming Cyanobacteria
Source: PLoS One. 2016 May 20;11(5):e0156034. doi: 10.1371/journal.pone.0156034 (PMC4874684; doi:10.1371/journal.pone.0156034)
Supplement: S2 File — Images providing depictions of specific interruption elements, element frequency relative to phylogeny, and element length and GC content trends. (PDF) [file pone.0156034.s004.pdf]

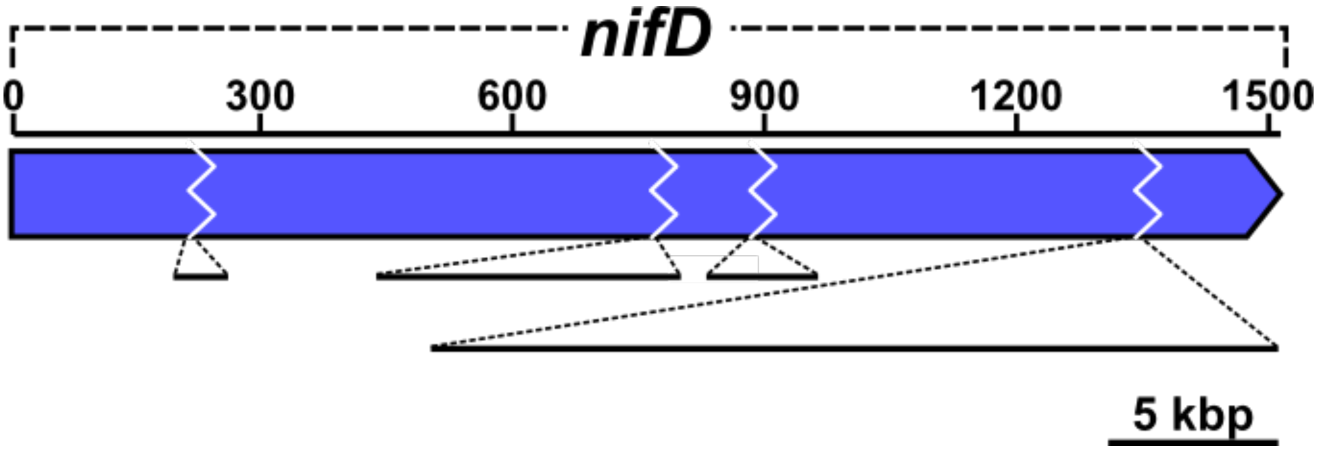

Figure A. Four elements within *Rivularia nifD*. The length of four elements interrupting the *Rivularia* sp. PCC 7116 *nifD* gene and their location within the gene.

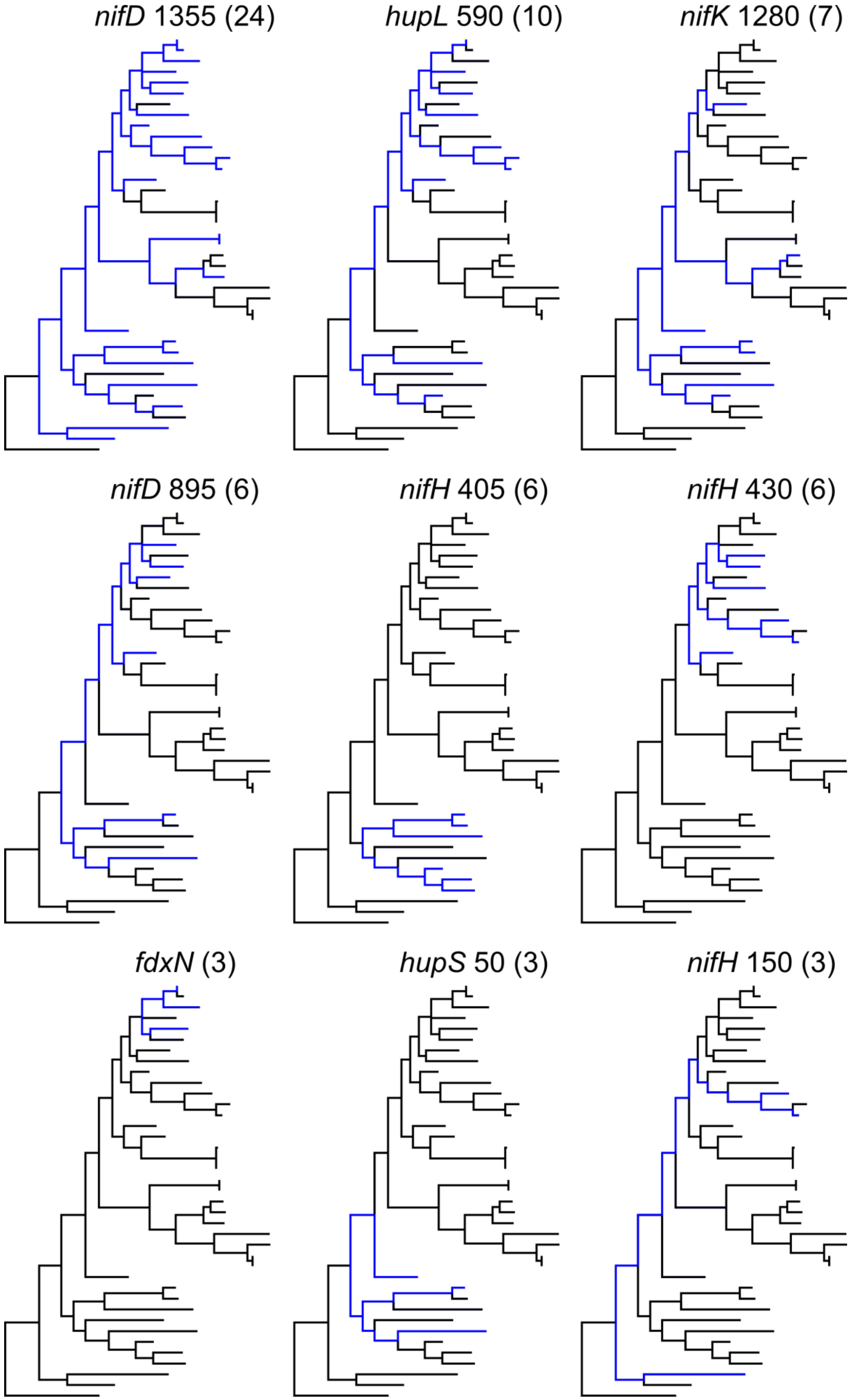

Figure B. Element distribution throughout 16S phylogeny. The presence of common interruption element variants (at least three representative elements) in heterocyst-forming cyanobacteria, in relation to 16S rRNA phylogeny (Fig 2). The headings are labeled as the interrupted gene, approximate position of interruption if that gene has multiple element variants, followed by the number of occurrences of that variant in the 38 genomes surveyed in this study. Blue lines and text highlight the organisms that possess each variant.

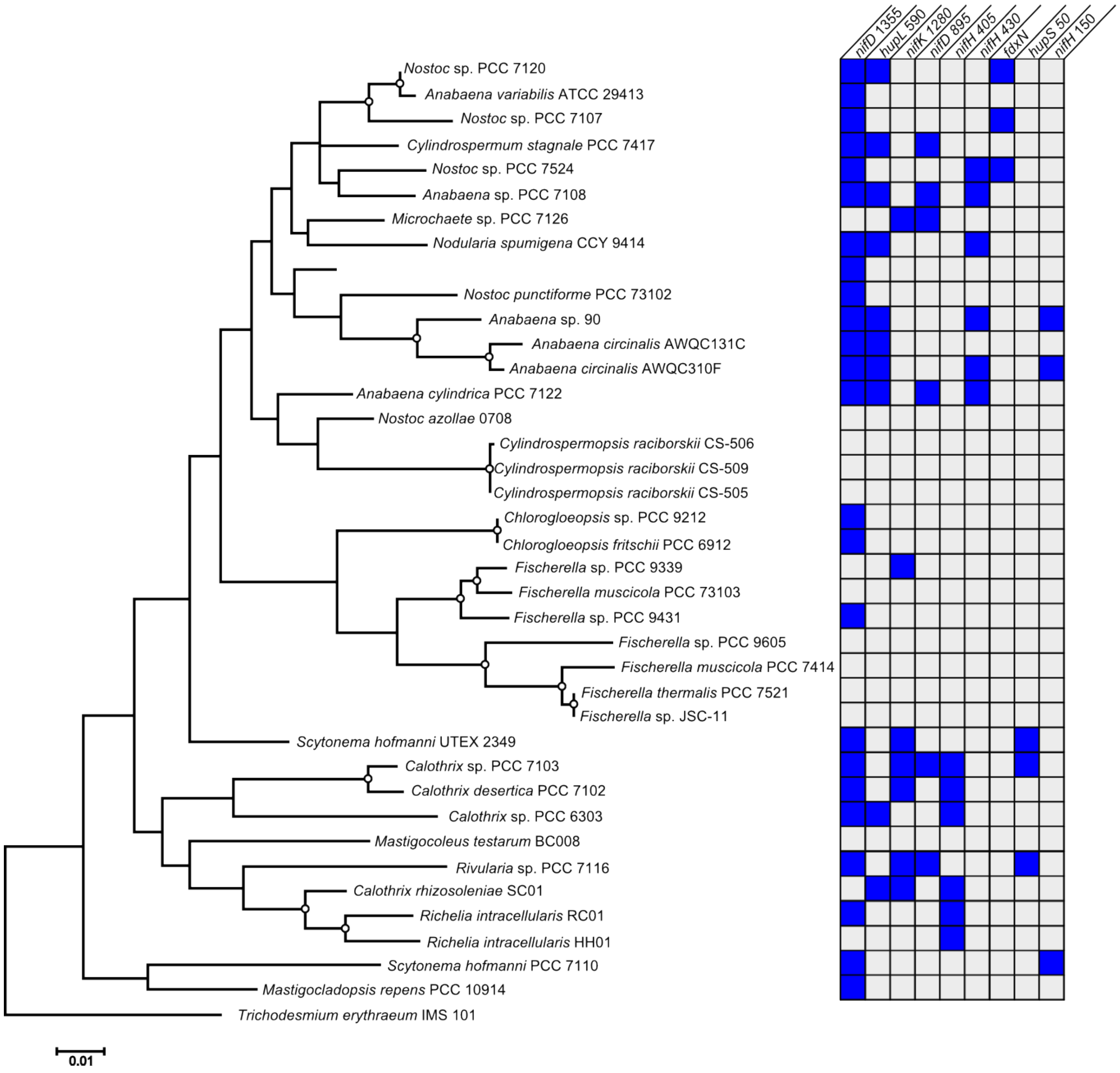

Figure C. Element distribution throughout 16S phylogeny. The same information that is displayed on the nine trees of S2 Fig, but condensed into a single tree with the organism names displayed. The element column headings are labeled as the interrupted gene followed by the approximate position of interruption if that gene has multiple element variants. Blue squares highlight the organisms that possess each variant.

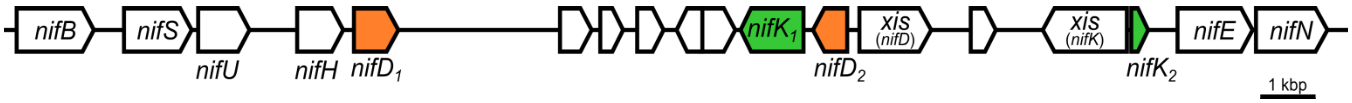

Figure D. *Fischerella* sp. PCC 9339 *nif* operon arrangement. The *nif* operon and surrounding genes of *Fischerella* sp. PCC 9339, showing interruptions within each the *nifD* and *nifK* genes, but with the two sections of these genes oriented in opposite directions, likely due to mis-assembly of the genome.

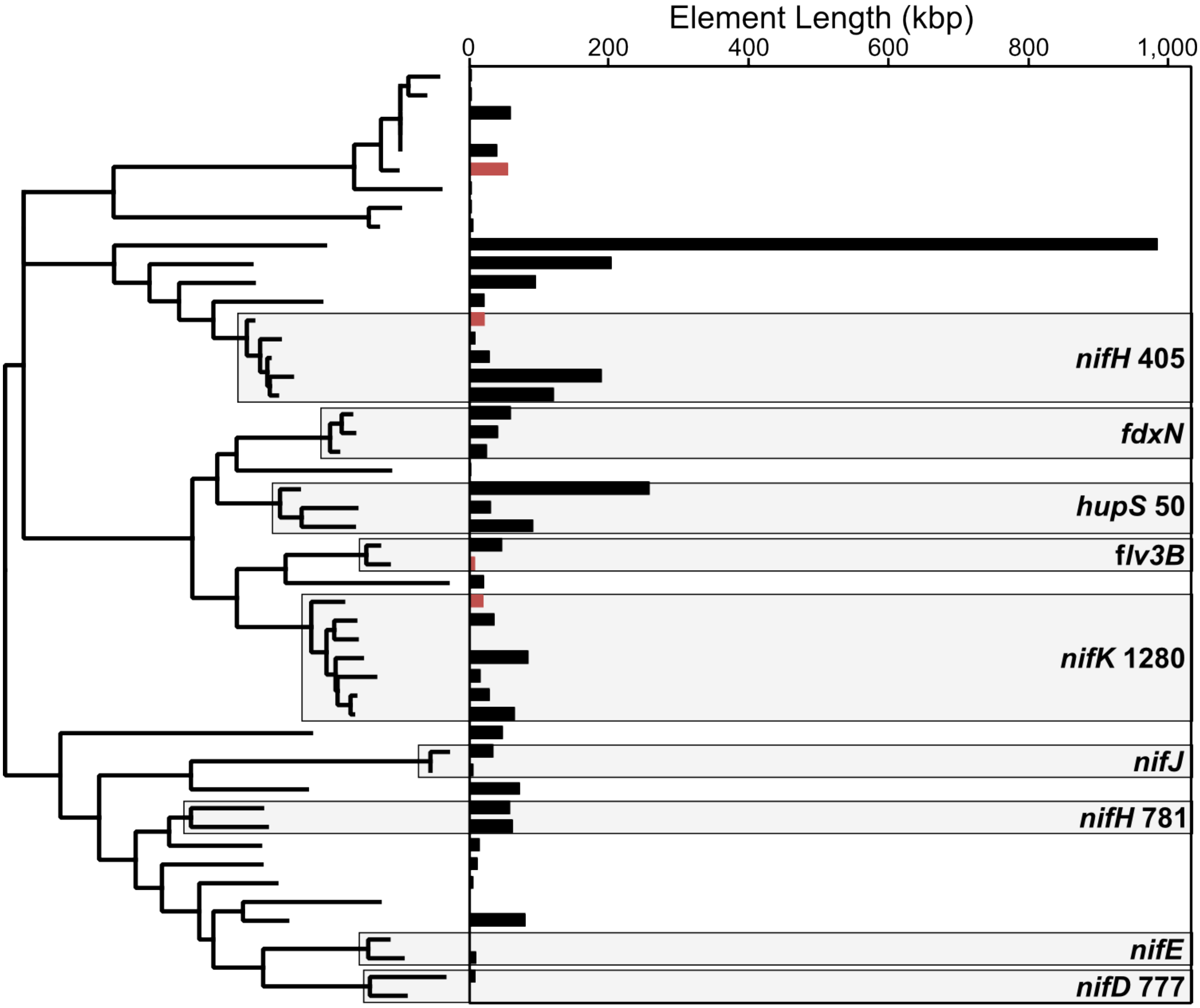

Figure E. Length of elements with serine recombinases. Maximum likelihood phylogenetic tree of serine recombinase gene (*xis*) nucleotide sequences (Fig 3) and the length of the element each *xis* gene is found on. Elements in red bars are not on a single contig, and thus, may be longer. Light grey boxes shade clusters of element variants and are labeled by the interrupted gene and, in cases where multiple variants were found for the same interrupted gene, the approximate location on the gene where the element was found.

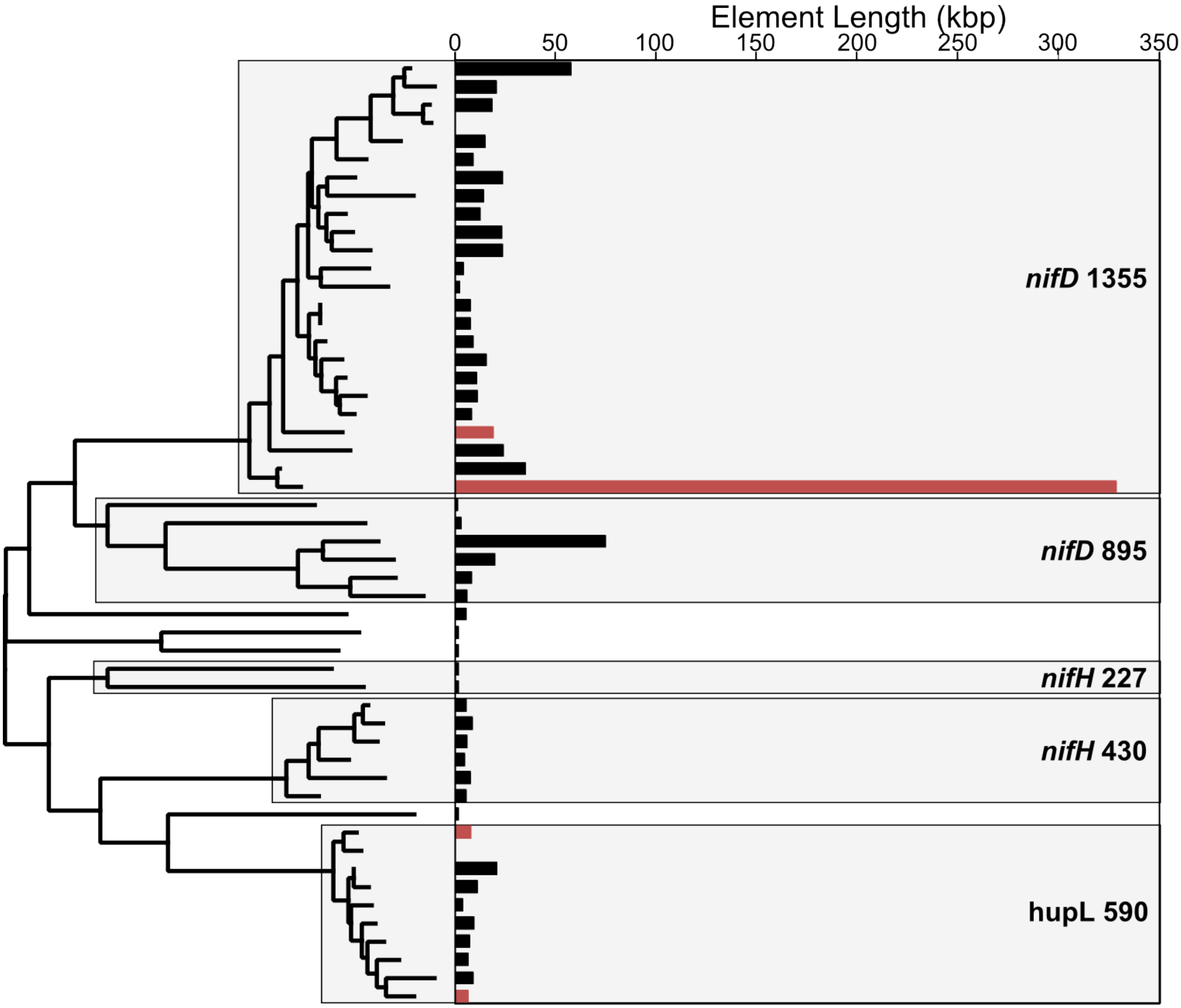

Figure F. Length of elements with tyrosine recombinases. Maximum likelihood phylogenetic tree of tyrosine recombinase gene (*xis*) nucleotide sequences (Fig 4) and the length of the element each *xis* gene is found on. Elements in red bars are not on a single contig, and thus, may be longer. Light grey boxes shade clusters of element variants and are labeled by the interrupted gene and the approximate location on the gene where the element was found.

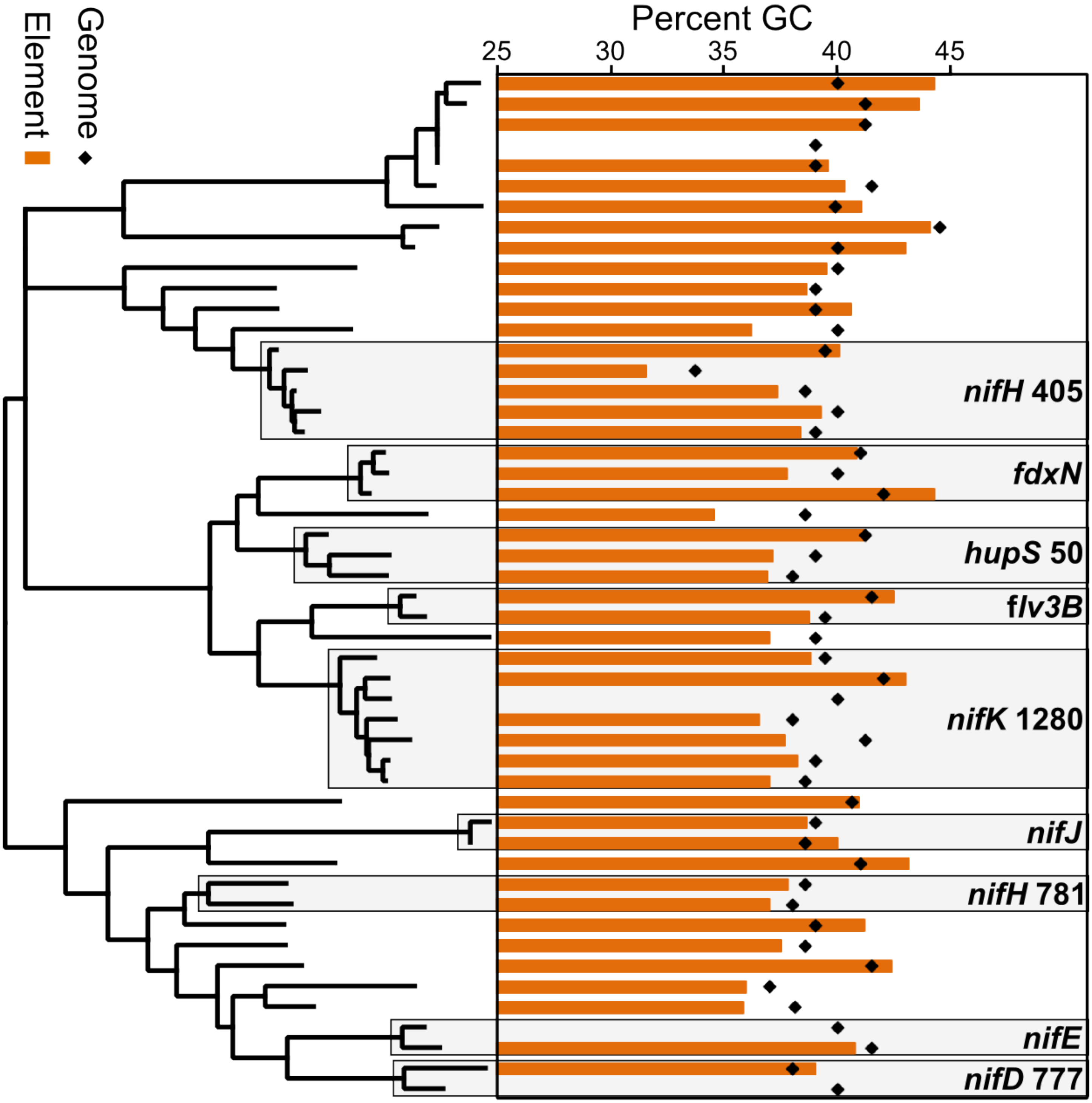

Figure G. GC content of elements with serine recombinases. Maximum likelihood phylogenetic tree of serine recombinase gene (*xis*) nucleotide sequences (Fig 3) and the GC content of the element each *xis* gene is found on (orange bar) and the genome it is in (black diamond). Light grey boxes shade clusters of element variants and are labeled by the interrupted gene and, in cases where multiple variants were found for the same interrupted gene, the approximate location on the gene where the element was found.

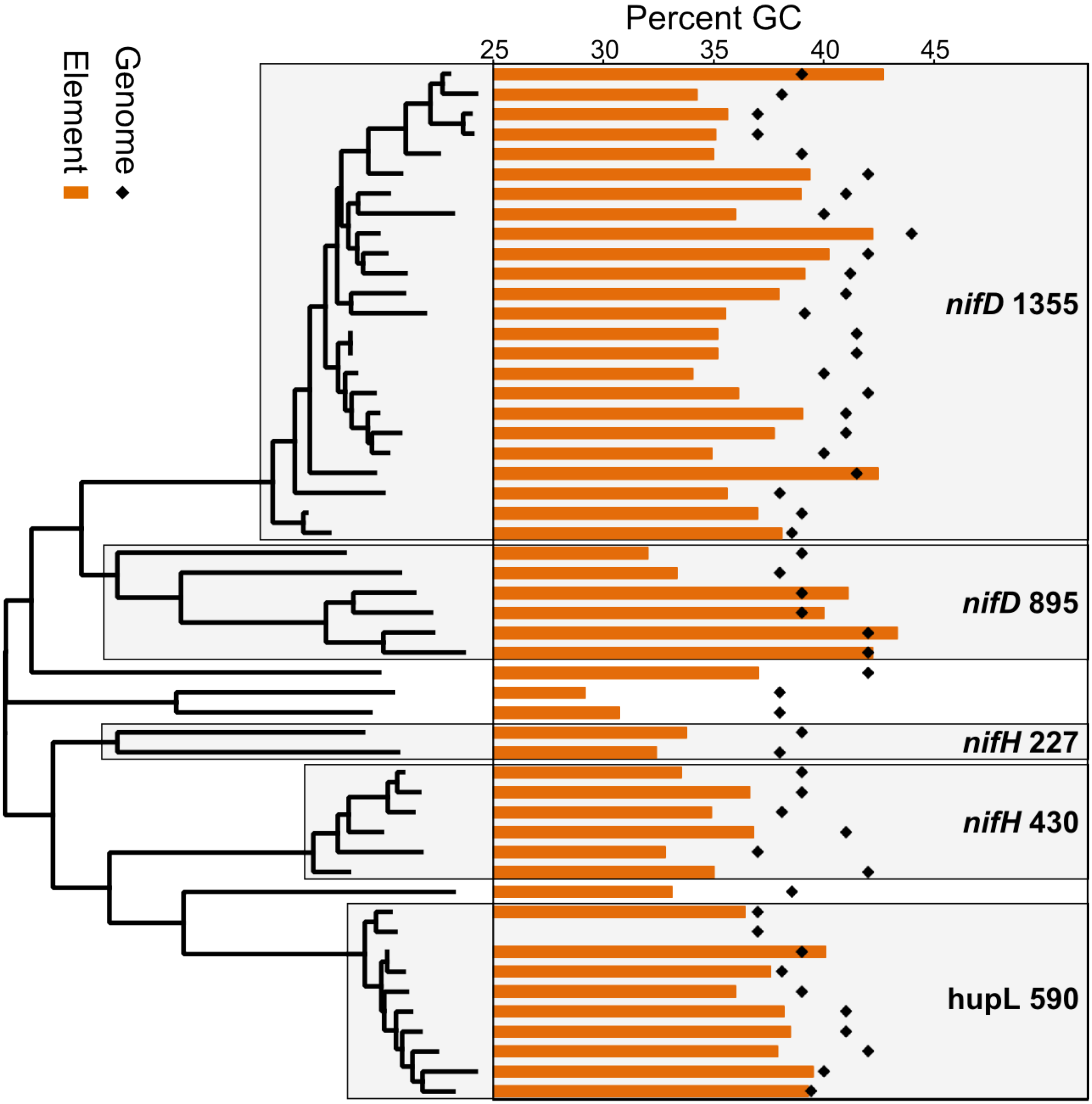

Figure H. GC content of elements with tyrosine recombinases. Maximum likelihood phylogenetic tree of tyrosine recombinase gene (*xis*) nucleotide sequences (Fig 4) and the GC content of the element each *xis* gene is found on (orange bar) and the genome it is in (black diamond). Light grey boxes shade clusters of element variants and are labeled by the interrupted gene and the approximate location on the gene where the element was found.
